# Study of Anterior Chamber Aqueous Tube Shunt by Fourier-Domain Optical Coherence Tomography

**DOI:** 10.1155/2012/189580

**Published:** 2012-06-21

**Authors:** Chunhui Jiang, Yan Li, David Huang, Brian A. Francis

**Affiliations:** ^1^Department of Ophthalmology, Eye & ENT Hospital, School of Medicine, Fudan University, 83 Fen-Yang Road, Shanghai 200031, China; ^2^Casey Eye Institute, Oregon Health Sciences University, 3375 SW Terwilliger Boulevard, Portland, OR 97239, USA; ^3^Doheny Eye Institute, Keck School of Medicine, University of Southern California, 1450 San Pablo Street, Los Angeles, CA 90033, USA

## Abstract

*Purpose*. This cross-sectional, observational study used Fourier-domain optical coherence tomography (OCT) to examine the position, patency, and the interior entrance site of anterior chamber (AC) aqueous tube shunts. *Methods*. The OCT, slitlamp biomicroscopy, and gonioscopy findings of 23 eyes of 18 patients with AC shunts were collected and compared. *Results*. OCT images demonstrated the shunt position and patency in all 23 eyes, and the details of the AC entrance in 16 eyes. The position of the tube varied, with the majority (14/23) on the surface of the iris. The exact position of the AC entrance relative to Schwalbe's line (SL) could be determined in 9 eyes (posterior to SL in 7 eyes, anterior in 2 eyes). At the AC entrance, growth of fibrous scar tissue was present between the tube and the corneal endothelium in all 16 eyes in which the entrance could be clearly visualized. It's a new finding that could not be visualized by slitlamp examination or lower resolution OCT. *Conclusion*. Compared to slitlamp examination, Fourier-domain OCT of AC tube shunts provided more detailed anatomic information regarding the insertion level relative to SL, scar tissue between the tube and the corneal endothelium, and patency of the tube opening.

## 1. Introduction

Anterior chamber (AC) aqueous tube shunt surgery improves intraocular pressure (IOP) control in glaucoma patients. Initially used as a treatment for refractory glaucoma, its use is now more widespread [1–3]. One of the possible complications of tube shunt surgery, however, is corneal endothelial cell loss which may lead to corneal decompensation [4–6]. The exact mechanism for this is unclear, with the leading theories being turbulent aqueous flow around the tube or intermittent touching between the tube and the cornea [4, 7]. In addition to the easily recognized direct touch of the corneal endothelium by the tube tip, the site where the tube enters the AC may also be a location of corneal endothelial contact and damage. In cataract surgery, the location of the incision is one of the factors that induce endothelial cell changes and decrease cell density [8, 9]. 

While there are many studies in which optical coherence tomography (OCT) has been used to image the anterior segment of the eye [10–14], only two have focused on aqueous tube shunts. In those reports, a time-domain OCT with limited resolution, 17 *μ*m full-width-half-maximum depth, was used to identify aqueous tube shunt patency and position in the presence of corneal opacities [15, 16]. Due to the relatively low resolution, fine structures such as the corneal endothelium and trabecular meshwork could not be visualized, and the level of the tube entry relative to Schwalbe's line could not be established [16]. In this study, we used a Fourier-domain OCT system with higher speed and resolution (5 *μ*m). The current study evaluates the position of the tube shunt relative to Schwalbe's line, as well as patency of the tube opening.

## 2. Methods

### 2.1. Data Collection

Patients that had previously undergone AC aqueous tube shunt implantation (Baerveldt Glaucoma Implant 350, Abbot Medical Optics, Inc., Santa Ana, CA, USA) by one surgeon (BAF) and were seen in followup at the Glaucoma Service of the Doheny Eye Institute (Keck School of Medicine, University of Southern California, Los Angeles, CA, USA) between July and September 2009 were included. Clinical data as well as slit lamp findings were collected from patient records, and gonioscopy was performed on all patients. OCT images of the shunts were acquired and analyzed and compared to slit lamp findings. Approval for data collection and analysis was obtained from the Institutional Review Board of the University of Southern California, and the research adhered to the tenets set forth in the Declaration of Helsinki.

### 2.2. Optical Coherence Tomography

Longitudinal imaging of the shunt was performed with a commercially available Fourier-domain OCT system, the RTVue (Optovue, Inc., Fremont, CA, USA). The system functions at 830 nm wavelength and is capable of 26,000 axial scans per second. It achieves a full-width-half maximum axial resolution of 5 *μ*m in tissue. The Corneal Adaptor Module-Long lens (Optovue, Inc.) was designed to provide telecentric scanning so that the OCT beam remains parallel to the central axis across the transverse scan range. This lens allows the acquisition of anterior segment images with a transverse resolution (focused spot size) of 15 *μ*m [17]. All of the images were acquired by a single ophthalmologist (CHJ).

High-resolution scan mode (1024 A-scans) was used with the scan line parallel to the axis of the aqueous tube shunt. The tissue scan depth was 2.0 mm, and the scan length was 6 mm. The OCT system acquired 16 line scans consecutively. These scans were registered, and the image frames were averaged to enhance signal strength and suppress speckle noise. 

### 2.3.  Measurement

Using the two-dimensional image recorded by the RTVue, the length of corneal endothelium covered by fibrous proliferation was measured with the software caliper tool provided by the instrument. It was measured between the anterior edge of the fibrous tissue on the corneal endothelium to Schwalbe's line. The distances were automatically calculated after these two caliper points were manually located.

### 2.4. Statistical Analysis

All data were analyzed using the Statistical Package for the Social Sciences software (version 13.0k, SPSS Inc, Chicago, IL, USA). Descriptive statistics were calculated as means and standard deviations. A Wilcoxon rank test was used to compare the followup period between patients in which the inner limbal entrance could or could not be visualized. The value of *P* < 0.05 was deemed to be statistically significant.

## 3. Results

### 3.1. Patient Enrollment and Demographics

The study cohort included 18 consecutive patients (23 eyes) who received AC aqueous shunt implants at the Doheny Eye Institute by one surgeon (BAF). The patients included 6 females and 12 males with a mean age of 70.8 ± 14.9 years (range: 37–89 years). Self-reported ethnic backgrounds included 8 Hispanic, 5 Caucasian, and 5 Asian. For the 23 eyes, 13 were diagnosed as primary open angle glaucoma (POAG), 5 as chronic angle closure glaucoma (CACG), and 5 as neovascular glaucoma (NVG). At the time of their visit, patients were using 2.0 ± 1.0 (range 0–4) topical glaucoma medications. With this treatment, IOP was less than or equal to 16 mmHg except in all but one patient, for whom it was 47 mmHg. The IOP ranged from 4 to 47 mmHg with a mean of 14.0 ± 8.4 mmHg. Visual acuity ranged from 20/30 to hand motion, with a median of 20/200. Optic nerve cup to disc (C/D) ratio was 0.88 ± 0.14 (range: 0.5 to 0.99). Six eyes were phakic, 16 eyes were pseudophakic, and one was aphakic. Conjunctival blebs were clinically evident in 19 eyes, and there was no bleb, or the bleb was unable to be visualized due to the upper eyelid in 4 eyes. The cornea was clear in 22 eyes, including 2 with a history of penetrating keratoplasty (PKP), and edematous in the one eye with 47 mmHg IOP and a history of PKP.

### 3.2. OCT Findings of the Aqueous Tube Shunt

**Figure 1:**
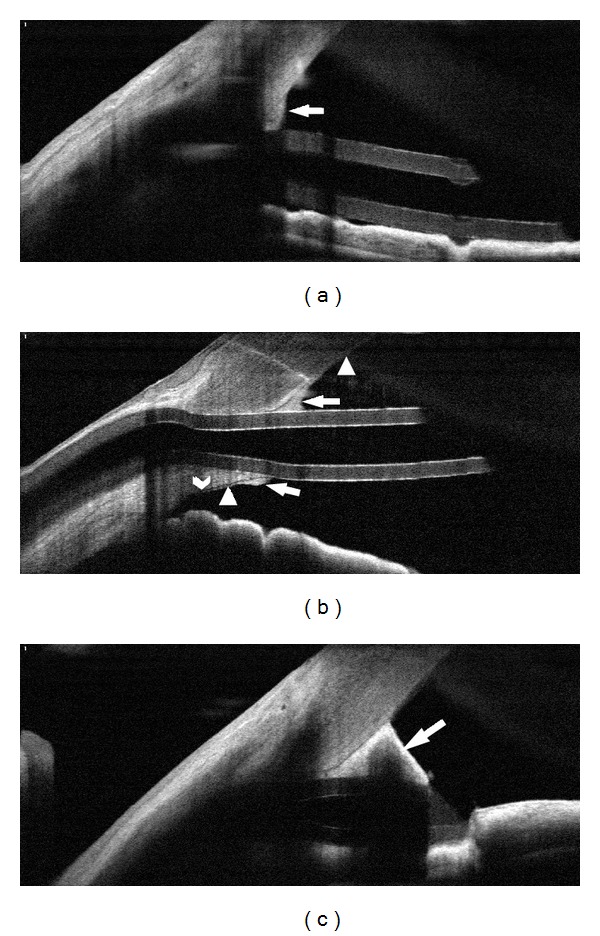


OCT imaging demonstrated the position and patency of the shunt in all 23 eyes. Details of the AC entrance were evident in 16 eyes, and the precise position of the tube shunt entry relative to the Schwalbe's line was visualized in 9 eyes. The position of the tube inside the anterior chamber varied. It was lying on the anterior surface of the iris in 14 eyes (Figure 1(a)), between the cornea and iris in 4 (Figure 1(b)), partially indenting the iris in 2 (Figure 1(c)), and disrupting the corneal endothelium at its entrance in 3 (Figure 1(b)). As visualized by OCT, the tube position was different from that determined by slit lamp examination in 2 eyes. Slit lamp biomicroscopy found one shunt close to the cornea and another touching the cornea, while OCT found that both tubes were well away from the corneal endothelium, between the iris and the cornea.

**Figure 2:**
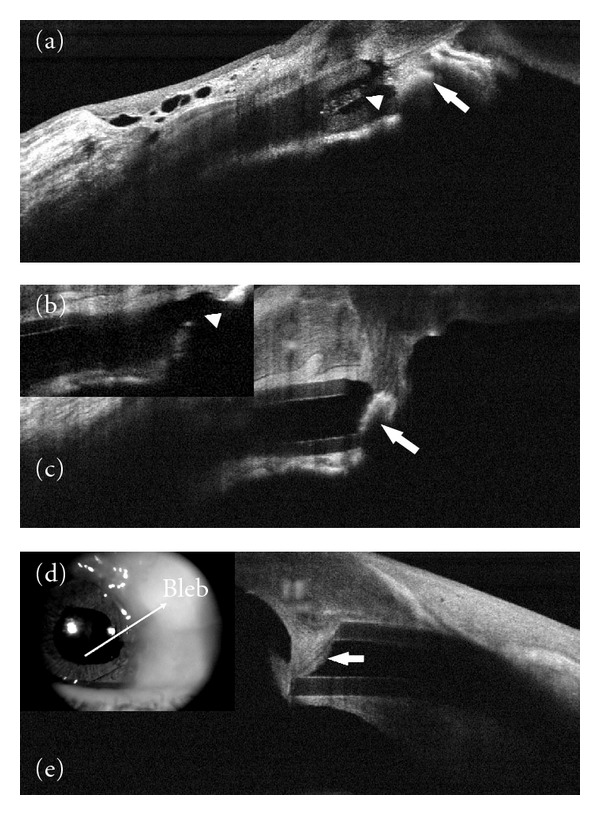


Based on OCT imaging, the tip of the shunt was patent (Figure 1) in 18 eyes, completely blocked (Figures 2(a) and 2(e)) in another 2 eyes, and partially blocked (Figure 2(c)) in 3 eyes. In the 4 of the 5 eyes with an abnormality at the tip, IOP was within normal range. It was extremely high, 47 mmHg, in the one where the tip was totally blocked. Compared to the other eyes, the 5 eyes with shunt tip abnormalities were not associated with high IOP (*P* = 0.539) or more glaucoma medications (*P* = 0.144). The incidence of abnormal findings was higher in CACG eyes than in POAG eyes (*P* = 0.034, Table 1). Both OCT and slit lamp examination detected tube blockage in the one case with extremely high IOP (Figure 2(a)). The other instances of tube blockage found by OCT were not detected by slit lamp examination and did not result in elevated IOP (Figures 2(c) and 2(e)). Compared to slit lamp biomicroscopy, 4 eyes had new findings by OCT including the tip indenting into the iris in 2 eyes, tip blocked in one, and partially blocked in another.

**Figure 3:**
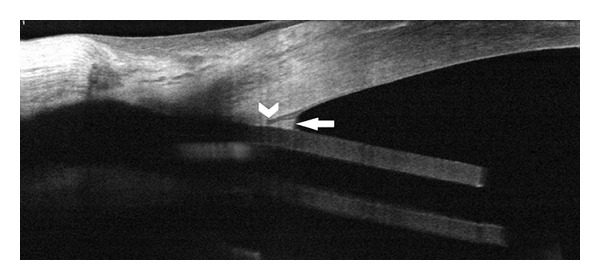


**Figure 4:**
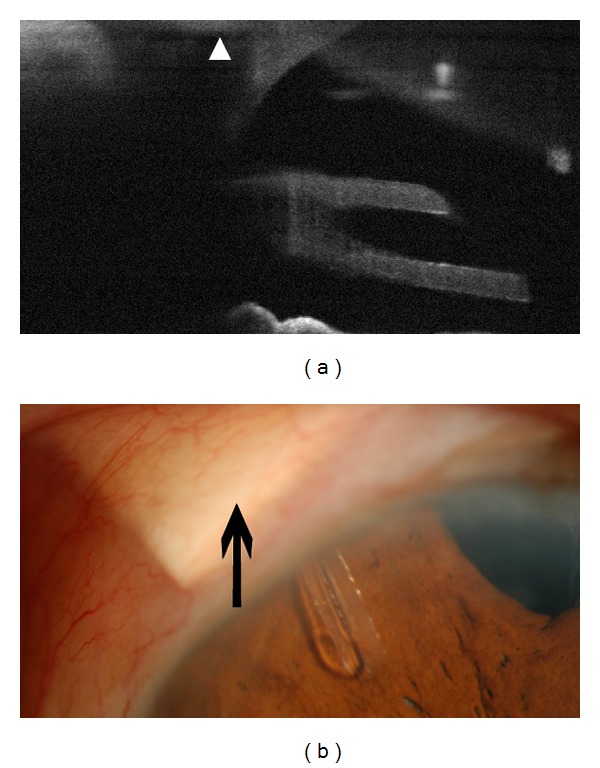


In 16 of 23 eyes, details of the inner entrance were evident by OCT, including the position of the tube shunt relative to Schwalbe's line (Figures 1(b) and 3) in 9 eyes. However in 7 eyes, OCT was unable to image the internal limbal entrance of the tube (Figure 4). Imaging of the inner entrance was apparently blocked by shadowing from the pericardium or scleral patch graft at the external limbus. These 7 eyes had a shorter average period (2.1 ± 1.6 months) between the implantation surgery and the study visit. For the other 16 eyes in which the inner entrance was imaged, the followup interval between surgery and imaging was 23.7 ± 23.6 months (*P* < 0.001).

In all 16 eyes in which the inner entrance was visualized (Figures 1 and 3), fibrous proliferative tissue was attached to the corneal endothelium around the inner entrance of the tube and, in some cases, the iris. In only one of them, this proliferation was recorded by the slit lamp as an unspecified white tissue at the level of the inner cornea or corneal endothelium. The clinical impression from the slit lamp exam was a corneal opacity as a sign of touching between the tube and the cornea and early corneal decompensation. However, the OCT showed that the tube entry was posterior to Schwalbe's line and that the observation instead represented fibrous tissue at the tube entry site.

The distance of corneal endothelium covered by proliferative fibrous tissue was 518.3 ± 291.0 *μ*m (*n* = 16; range: 251–1130 *μ*m). The relative position of the tube shunt was posterior to Schwalbe's line in 7 eyes and anterior to it in 2 eyes (Figures 1 and 3). There were no differences in the IOP and glaucoma medications with regard to the positions relative to Schwalbe's line (*P* = 0.586 and 0.540, resp.).

## 4. Discussion

In this cross-sectional observational study, we analyzed the OCT images of 23 eyes with aqueous tube shunts to study the details of the position, patency, and the inner entrance of the tube. In our study, the use of high-resolution 830 nm Fourier-domain OCT enabled the visualization of the fine structural details of the inner entrance of aqueous tube shunts in the anterior chamber. To our knowledge, this is the first study of this kind. In many cases, the location of the inner entrance could be localized to either anterior or posterior to Schwalbe's line. Entrance anterior to Schwalbe's line was associated with disruption of the corneal endothelium. In addition, fibrous proliferative tissue attaching the tube to the corneal endothelium around the entrance was present in all eyes.

In our analysis, most tubes were clear from the cornea and lay on or near the surface of the iris. The mechanism for corneal decompensation with aqueous tube shunts is not fully understood, but endothelial contact at the entry site may be a risk factor [4, 7]. A previous study showed that the distance between the tube and the cornea was not related to the dropout of the endothelium [18], but intermittent touching of the corneal endothelium due to blinking and rubbing of eyes cannot be ruled out [19]. However, previous studies in phakic intraocular lens patients showed that corneal endothelial dropout is related to the distance between the lens implant and cornea, and they also showed intermittent touching of the corneal endothelium by the intraocular lens [20–22]. These patients were highly myopic and tended to have a deeper anterior chamber than many glaucoma patients, and the shunt is more flexible than the intraocular lens. So placement of the tube as far from the cornea as possible is likely to be important, with the best location near the surface of the iris. Using OCT, the distance between the tube or tube tip and the corneal endothelium can be measured but is sometimes difficult due to the limited scan range of 1.8 mm with RTVue-CAM. Further study with long-term followup is required to draw a correlation between the tip to corneal endothelium distance and endothelial cell dropout.

The occlusion of the tube tip has been reported by others [23]. Theoretically, if the aqueous outflow was blocked, there should be no posterior filtering bleb. However, in one of our two cases with apparent total blockage, the bleb was still present and the IOP was controlled, indicating that there must have been a small communication at the tip of the tube. In the case with partial blockage, one OCT scan showed the tip completely blocked by a membrane while another scan showed that a small cleft was present in the membrane that allowed the outflow of the aqueous. Thus, it is important to acquire more than one longitudinal cross-sectional scan to avoid missing such important information. The 3D mode might be helpful in such cases, as it takes a series of scans instead of one. Alternatively, the physician him/herself may need to perform the scan to see the real-time display of many sections through the tube rather than just look at one section recorded by a technician.

Because of the high resolution of OCT, the fine structure of the inner limbal entrance was also imaged in our study. The entrance in patients with a longer term of followup was more easily visualized. This may be related to the pericardium or scleral patch graft placed on the external surface of the sclera adjacent to the limbus. Due to its high density, it blocks the transmission of light that is used in OCT exam. This tissue will thin and be partially absorbed with time, so the entrance in patients with a long-term followup is more likely to be visualized. To avoid corneal endothelial trauma and possible corneal decompensation, the entry site of the tube should be posterior to Schwalbe's line.

In all 16 eyes in which the entrance could be clearly visualized, some proliferative tissue was present around the tube entrance, attaching the tube to the corneal endothelium. This proliferation may cause additional damage via traction on the endothelium or by blocking the nutritive elements of aqueous from reaching the corneal endothelium. It is also important to differentiate this finding from a corneal opacity that may represent touch points between the tube and the cornea and early decompensation.

There were several limitations to our study. The first was its small sample size. Despite the many types of AC shunts implanted clinically, only one kind of aqueous shunt was studied here. As this was a cross-sectional study and corneal endothelial density was not recorded, the relationship between the endothelial density and the OCT findings was not evaluated. Thus, further study with long-term followup and more cases may determine the significance of the OCT findings, including the position of the tube, location of the entrance, and presence of fibrous tissue proliferation.

In summary, high-resolution Fourier-domain OCT was used to evaluate the position of aqueous tube shunts in the anterior chamber relative to the cornea and iris in all patients. For patients with longer-term followup, details of the inner limbal entrance were discerned. High-resolution OCT may be useful in assessing tube patency, especially if occlusion is suspected clinically. It may also be helpful to study corneal complications by showing if the entry site is anterior or posterior to Schwalbe's line and providing measurements of fibrous tissue along the endothelial surface and the distance between the tube tip and endothelium.

## Figures and Tables

**Table 1:** Incidence of abnormal findings at shunt tips in different types of glaucoma.

	POAG	CACG	NVG
Abnormal cases	0	4^∗^	1
Total cases	13	5	5

POAG: primary open angle glaucoma, CACG: chronic angle closure glaucoma, NVG: neovascular glaucoma. *P = 0.034 compared to POAG.
